# "The Hospital" Medico-Sociological Supplement (No. II)

**Published:** 1911-02-11

**Authors:** 


### "THE HOSPITAL" MEDICO-SOCIOLOGICAL SUPPLEMENT.

(No. II.)

### GENIUS AND DECADENCE.

Artists and women, says Nietzsche, hate truth: the reception the medical scientist gets when he ventures to claim a small share in the criticism of art or literature inclines one to think that half, at any rate, of the apophthegm is true. We believe a fairly strong case has been made out for the statement that certain painters were really a little colour-blind; and one of the school of Major Ronald Ross wrote a treatise arguing that the fading of the splendour of Greece might be partly imputed to the anophelinæ. There is to the medico-scientific mind a pleasant disillusioning smack about both these theses, but the world's temper is more romantic. It likes, for instance, Mr. Kipling's reasons for the white man swamping the aboriginal much better than Dr. Archdall Reid's. What now concerns us, however, is not the predilections of the multitude but rather the ignorance or cowardice of the learned—to be particular, the failure of critics to deal faithfully with the morbid side of certain literary artists' work.

### The Lop-Sidedness of Genius.

Some months back a medical contemporary noticed, with an ably expressed mixture of disgust, scorn, and amusement, an audacious book by a distinguished living minor poet in deliberate praise of sexual inversion. Now it is coming to be perceived that, in common parlance, genius must be paid for. The man of genius is a lopsided person. He has hypertrophied intellect and creative power, but makes up for this, as it were, by grave deficiencies in other directions. He is, much more frequently than the ordinary man, a chronic invalid, or alcoholic, or epileptic, or a moral idiot. Commonly enough he becomes insane. Writers who sneer at Lombroso and (but less often) at Nordau have never made (much as it is to be wished that they could make) anything like a satisfactory contradiction of this proposition. It is again discouraging, but still diverting, to see how leading critics miss the significance of facts like these, naïvely regretting the o'er-informed tenements of Pope, Leopardi, Keats, and Chopin, and the contrast between Rousseau's precept and his practice, or strenuously whitewashing Burns and Byron.

### The Dearth of All-Round Men.

There are several reasons for this. In this country men of all-round acumen, like the late Grant Allen, rarely occur. As Mr. Herbert Paul well says, ordinary persons of intellectual talent soon sort themselves out into the literary and the scientific, two camps between which there is small communication. Then, too, it must be remembered that the whole subject going by the name of Degeneracy, perhaps in consequence of having been badly handled by its later exponents, has never received the faintest real notice from orthodox British science. Last, but not least, there is the optimistic national temperament, the British instinct, of vast natural strength, to put a good face on things whatever they are. But one would think that in spite of these disabilities the cloud of witnesses should make an impression on the commonsense of here and there a one.

### The Example of Walt Whitman.

Here, at any rate—put as briefly as possible—is an example of what we mean. Walt Whitman's is a name held in considerable honour. His altruism and self-disinterestedness and wide human sympathy, with his talent, have made him a reputation throughout the Western world. The only thing said against him is that the form of his message is crude and without beauty. But there is not the least doubt at all that he preached insistently to his fellow men, not altruism and democracy alone, but something else too; and that something else is homosexualism. There are many who, when confronted with a plain statement like this, contradict first and think and examine afterwards. To such it may be recommended to read *Recorders Ages Hence*, *What I Heard at the Close of Day*, *A Glimpse*, and particularly the concluding part of *So Long!*; and then to compare what they have read with the dismal, ridiculous confessions to be found in, say, Dr. Moll's *Untersuchungen über die Libido Sexualis*. There can be only one conclusion.

### The Common-Sense Conclusion.

What should be done about it now is, perhaps, not quite as obvious. The ordinary reformer, thinking, as ordinary reformers do think, only of society, would advise that from all popular editions of Whitman's works these rotten exuberances should be excised; and it is at all events worth while pointing out that a well-known book like *Leaves of Grass* violates the usual canons governing publication, in a startling way. But after all these passages can only do harm to those predisposed to the tenets expressed in them. The usually received verdict is that the sexual pervert is more mad than bad; and the sooner his madness comes to light and he is restrained the better for the race; because, as authorities like the one quoted agree, the morbid attraction is not always exclusive, and sterility cannot, unfortunately, be counted upon. But it should not be possible, as it now is, to say this in the case of the convicted offender.

### ALCOHOLISM AND DEGENERACY.

That drunkards are degenerate and are the parents of degenerates is a truism that threatens to become a platitude. There is no speaker on the temperance question who cannot point to the sickly, feeble-minded, and often criminal children of drunken parents. Nor can the fact be doubted. The children of alcoholists—to use a convenient word coined by the Eugenics Society—are as a rule poor creatures. But the question arises, Of what nature and temperament are, apart from alcoholism, their parents? To put it briefly: "Is alcoholism a disease in itself, of which one of the symptoms is degeneracy of the offspring; or is alcoholism in itself a symptom of degeneracy?" At present we have so little control of the feeble-minded that they almost never come within the purview of the social observer until they have been guilty of some fault which brings them within reach of the law. We have thus the habitual inebriate, the thief who begins stealing the day after he is released from prison—like Mr. Lloyd George's shepherd—the creature whose immorality cannot, apparently, be controlled by any punishment that may be inflicted. Pathologically regarded, these are all degenerates, and their children are likely to inherit the characteristics of the parents; but, failing a clear life history of each criminal—which is in most cases unattainable—it is difficult to say whether the degeneracy is the result of the crime or the crime the result of the degeneracy.

### Crime and Alcoholism.

The statistics of crime in general, and of alcoholism in particular, are as yet new and incomplete, and they have in many cases been compiled with a view rather to support a theory than to elucidate a fact. That of the degeneracy of the children of alcoholic parents is a case in point. From this point of view some statistics collected by the Francis Galton Laboratory of National Eugenics are valuable, because they are not put together to support the cause of either alcoholic indulgence or total abstinence. Unfortunately they deal with only a small number of cases, and those only of females; but in face of the statement, so often made at temperance gatherings, that while men are becoming more sober, women are becoming more drunken—a statement never based on any statistics—this study of women alcoholists may be of value. The total number dealt with is 207, and are women who have been sent to Langho, the Lancashire Reformatory for Inebriates. Of course it must be remembered that persons who get into an inebriate reformatory are the extreme cases of alcoholism, and that in most cases they drift there because they have no one left with sufficient sense either of affection or of duty to save them from being convicted of drunkenness in a police-court. Drunkards, either men or women, belonging to the richer classes do not come to the reformatory; they have friends who may seclude them, but will take care to avoid an open scandal. Of the women alcoholists who have been sent to this reformatory a great majority are prostitutes. Allowing for the causes that lead normal women to prostitution—vanity, laziness, and occasionally seduction—most social workers know that a great number of the women who follow this mode of life are feeble-minded. It is quite conceivable that the life of the prostitute leads to alcoholism, and that this in turn may lead to alcoholic dementia, but an experience of "rescue homes" filled by young women who would, if they had not been thus saved, infallibly have drifted into prostitution, shows that a very large proportion of them are feebleminded—not aggressively imbecile, but "simple"; decidedly below the normal in intelligence and power of self-protection. A few years of loose living would soon drive them to drunkenness and in all probability to a reformatory. But the mental weakness precedes both forms of degeneracy, and is the cause of both. It is more difficult to trace the working of alcoholic degeneracy in the case of men, because these are more rarely sent to a reformatory—magistrates having regard to the fact that a man, at the worst, does something towards supporting his family, and therefore, as a rule, letting him off with a fine or a short term of imprisonment. But where there is pronounced, inveterate alcoholism, it will usually be found that the victim has previously shown some form of mental weakness, sometimes criminal, sometimes merely emotional.

### Inaccurate Speculation *versus* Scientific Investigation.

Therefore the inference that, because the children of such alcoholists are defective, the defect is due to their parents' drunkenness is rash, and often inaccurate. The chances are that the parent as well as the child is defective, although—social study being but a recent thing—the defect was never noticed until it showed itself in violent alcoholism. Trace back the life history of the alcoholic woman, and it will usually be found that even in her childhood she was morbid, lachrymose, hysterical, and that these characteristics became more marked after puberty. Pain during the menses would be the first excuse for alcoholic indulgence, but throughout there would be evidence of lack of self-control, that failure of balance which leads to alcoholism in one case and to asceticism in another. Trace back the life history of the alcoholic man, and you will generally find that he was always irritable, impatient of control, ready to argue about trifles and to throw up work, even when in want of daily bread, for some fantastical point of honour or suspicion of insult. Again, a lack of balance and self-control! And it is just such natures that are prone to indulge in alcohol when their nerves are upset. The alcoholism is a result, not a cause of the nervous excitement. That the children of such parents should exhibit defects of mind, nerves, and temper, is only to be expected, and to set all their defects of mind and body down to alcoholism on the part of one or both parents, without inquiring into the predisposing causes of the alcoholic tendency, is to leave the question half unanswered. Abnormality descends from parent to child, whether the abnormality takes the form of direct hereditary degeneracy, as is most common, or violent reactions, such as make the son of the agnostic a revivalist preacher, the son of the drunkard a passionate teetotaler, and conversely the son of the evangelical an atheist, and the son of the abstainer a sot. Whatever form it may take, these emotional developments show excess—lack of balance.

### Mental Deficiency.

But to set down all mental deficiency to alcoholism in the parent is quite unreasonable. It might fairly be argued that we are all descendants of hard drinkers. The great-grand-parents, if not the grandparents of most of us consumed—unless the records of their times are wholly false—an amount of alcohol that would frighten the hardest-headed of their descendants. The glory of the three-bottle man has departed, and feats of consumption which were held to be rather creditable than otherwise, even in the middle of the last century, would now drive any one who imitated them out of decent society. But if drinking by itself caused degeneracy in the offspring most people would be degenerates. As a matter of fact, the hard drinking of a healthy, non-neurotic generation did but little harm to their descendants. If a drunkard's son proved to be imbecile, insane, or criminal, it is necessary to go beyond the father's drunkenness to the characteristics he showed apart from alcohol, and these will almost certainly show points of degeneracy which, expressed only in alcoholism in him, find other outlets in his son. Alcoholism, in short, is a symptom of degeneracy rather than a cause of it. This does not mean that we should let the drunkard go unheeded to his doom, for he will assuredly injure both himself and those belonging to him in his course. But it does imply that we should study most carefully those who depart from the normal balance of mind, and in cases which tend to the extreme assume more power over their future than the law at present permits us to do.

